# Tocophobia experience and its impact on birth choices among Nigerian women: a qualitative exploratory study

**DOI:** 10.11604/pamj.2021.39.282.27229

**Published:** 2021-08-31

**Authors:** Deborah Tolulope Esan, Oluwaremilekun Christiana Thomas, Opeyemi Adeniyi Adedeji, Agatha Ogunkorode, Isaiah Dada Owoeye

**Affiliations:** 1 Department of Nursing Science, College of Medicine and Health Sciences, Afe Babalola University, Ado-Ekiti, Nigeria,; 2 Obstetrics and Gynecology Department, University College Hospital, Ibadan, Nigeria

**Keywords:** Tocophobia, Nigerian women, birthing choice, maternal request, primigravida

## Abstract

**Introduction:**

while pregnancy is a very beautiful and memorable experience for most women, some women may experience apathy towards childbirth and have feelings of fear and anxiety (tocophobia). Tocophobia can be far reaching with adverse physiologic and emotional consequences for mothers, infants and families. This study therefore explored tocophobia experiences among primigravid women and explored its influence on birth choices among antenatal women.

**Methods:**

the study employed a qualitative exploratory research design. Participants who were primigravida (women who had never experienced childbirth), were selected using purposive sampling technique on antenatal clinic days. Data was obtained using semi-structured interview questions. Data was analyzed using content analysis approach and thematic categorization.

**Results:**

results showed that few of the women experienced tocophobia and these fears were not strong enough to make them opt for caesarian section. Reasons attributed to tocophobia experience among the participants included “horror stories” told in the neighborhood, “past experiences” of close acquaintance and “entertainment videos” broadcast. Furthermore, all the participants preferred to have vaginal delivery.

**Conclusion:**

few of the primigravid women in Ekiti State, Nigeria, experience tocophobia and this experience does not influence their choice of delivery option, as all participant´s preferred vaginal delivery to caesarian section. It is important for midwives to be aware of their role in counselling and identifying women with tocophobia in order to promote good and safe transition from pregnancy to motherhood.

## Introduction

The word “tocophobia” comes from the Greek word *tokos*, meaning “childbirth” and *phobos*, meaning “fear”. Tocophobia is an intense anxiety or fear of pregnancy and childbirth, with some women avoiding pregnancy and childbirth altogether [1]. The word *“Tocophobia”* is often used interchangeably with fear of childbirth (FOC). Pregnancy is a beautiful and memorable experience for most women and also a point of transition in a woman's life to becoming a mother. Unfortunately, while many women embrace this happy moment and look forward to this experience, many also have apathy towards childbirth and experience feelings of fear, anxiety, unhappiness, loneliness [2]. Studies have shown that fear of childbirth affects about 20% of women in the world [3]. This fear is related to labour process and pain, fear of the physiological and physical changes that the woman would undergo and can cause women to avoid pregnancy altogether.

There are certain levels of fear and anxiety about childbirth that are expected, especially among primigravid mothers. However, it becomes a problem when these feelings negatively influence a woman´s decisions and perceptions about the birth process [4]. Fear of childbirth is also known as fear of vaginal delivery [5]. Childbirth-related fear (CBRF) has been described as a negative reasoning of the expected childbirth, feelings of fear and anxiety when facing birth, negative feelings towards childbirth and the unreasonable dread and avoidance of childbirth. Contrary to the widely reported aversion to caesarean section in the West African sub-region, Maternal Demand for Caesarean Section (MDCS) seems to be on the increase, and there is little evidence to explain this trend. The first study on MDCS published from Nigeria and West Africa was conducted amongst south-eastern Nigerian women requesting MDCS between 2003 and 2006. In that study, 4.4% of all MDCS deliveries were due to maternal request [6].

There are so many reasons that have been reported for fear of childbirth such as fear of pain, fear of losing control, fear of rupturing, fear of vaginal tear, fear of episiotomy, fear of pelvic floor damage, fear of uterine organ prolapse, fear of faecal and urinary incontinence, fear of operative delivery and fear of having an impaired or stillborn infant. A number of factors have been linked to the increased prevalence of fear of giving birth, such as young maternal age, advanced maternal age, nulliparity, preexisting psychological issues, inadequate social support and a history of abuse or difficult obstetric procedures [7]. Fear of childbirth has effects on women´s health as it is a major psychological issue that promotes women´s requests for medical interferences and interruption of physiological labour. If a pregnant woman thinks that she is not able to handle normal delivery, the resulting fear and anxiety makes her prefer cesarean section even in the absence of medical or obstetrical reasons. Such fears may also interfere with mother-child bonding and it is common knowledge that morbidity is higher in cesarean delivery.

Previous studies have indicated that the antenatal presence of tocophobia i.e. fear of childbirth leads to an increased risk for the pregnant woman having a negative experience during the birth [2,3]. A woman´s FOC (fear of childbirth), especially intense fear, influences how the pregnancy proceeds, the course of the birth and postpartum bonding [8]. This study therefore seeks to explore tocophobia experience among primigravid women attending a tertiary health institution in Nigeria and the influence of this on the birth option plan.

## Methods

This study used a qualitative exploratory research design. The study was carried out in a tertiary health institution in Nigeria (Federal Teaching Hospital, Ido-Ekiti, Ekiti State). The hospital is located in a sub-urban area in Ido-osi Local Government Area of Ekiti State, Nigeria. This government owned hospital is a 280-bed tertiary institution formerly known as Federal Medical Centre, Ido-Ekiti. The hospital was established in July, 1998 as a Federal Medical Center and later upgraded to a teaching hospital in September, 2014 by the Federal Government of Nigeria. The hospital serves as a referral centre for all other health institutions in Ekiti State. This hospital has 24 fully functioning departments comprising of 18 clinical departments including obstetrics and gynaecology department and 6 supportive departments. It has a capacity of 280 beds spanned through the following wards: male surgical, female surgical, male medical, female medical, paediatrics, accident and emergency, psychiatric, obstetrics and gynaecology and neonatal wards, surgical and medical outpatient´s department inclusive. It also has functional renal unit, cardiac unit, intensive care unit, ear, nose & throat (ENT) and ophthalmology units.

Averagely, the obstetrics and the gynaecology department attends to about 500 patients monthly. The department conducts antenatal clinic every Tuesday and Thursday morning. It conducts antenatal booking clinic every Monday morning, conducts family planning clinic every day and conducts postnatal clinic every Friday morning.

The target population included all women of childbearing age who were pregnant for the first time (who had never experienced childbirth) and who were attending Federal Teaching Hospital, Ido-Ekiti antenatal care clinic. The study was conducted between December, 2018 and January, 2019. Non-probability purposive sampling was used in order to meet the specific criteria of the study. The sampling criteria for the selection of participants were as follows: 1) women of reproductive age; 2) women who were pregnant for the first time; 3) women attending antenatal clinic. Participants were interviewed during clinic visits, where the researcher explained the study and obtained informed consents before the commencement of each oral interview. Participants were informed that the research was voluntary and that they were free to terminate the interview at any time during the research process. Face-to-face, semi-structured interviews were used to obtain data from the participants. Data collection focused on themes about fears related to childbirth, effects of fears on preference for caesarean section and preferred method of delivery. The questions were aimed at identifying the fears associated with childbirth, determining the effects of the fears on the women´s preference for caesarean section. Open-ended questions were designed to account for such flexibility in line with the focus of the study. This method was employed to initiate a conversation between the researcher and the respondents in order to explore the subject in detail. Each interview took about 20 to 30 minutes. The sample size was determined by saturation of data which was realized as 14 participants were interviewed and 3 more participants were interviewed to ensure referential adequacy, making a total of 17.

The interviews were voice recorded with the permissions of the participants and were transcribed verbatim except where translations were imperative. Interviews were conducted in one language: English as participants were asked prior to the start of the interview if they understood English. Interviews were transcribed, carefully read and double-checked for accuracy by the researcher. Data were analyzed and themes were identified. Analysis was based on content analysis, which involved organizing and categorizing evolving concepts systematically under the identified themes. The data were manually content analyzed.

Ethical clearance was obtained from the Ethics and Research Committee of Federal Teaching Hospital, Ido-Ekiti, Nigeria (Protocol Number: ERC/2018/11/26/164B). Informed consent was obtained from participants before commencement of data collection. Participants were informed about the purpose of the study and confidentiality was also maintained.

## Results

**Socio-demographic characteristics of participants:** a total of 17 women who were primigravid, between 15 and 33 years of age, participated in this study. As shown in Table 1, all participants were Christians with majority (88.20%) being Yoruba. Majority (88.20%) of participants were married, while very few (11.80%) were single. Most of the women (47.06%) were in their third trimester, some (35.30%) were in their second trimester while a few (17.60%) were in their first trimester. Majority (88.20%) of participants had undergone tertiary education while few (11.80%) have an educational level of secondary school. Seven of the participants at the time were not earning any wages or salaries, with 3 students and 4 unemployed. Five of the participants had previously had a miscarriage.

**Table 1:** socio-demographic characteristics of participants (n=17)

ID. No.	Age	Tribe	Religion	Occupation	Educational status	Marital status	Gestational age (months)	Previous miscarriage
1	29	Yoruba	Christian	Teaching	Bachelor's degree	Married	8	None
2	28	Yoruba	Christian	Civil servant	NCE	Married	3	None
3	27	Yoruba	Christian	Student	NCE	Married	8	None
4	30	Yoruba	Christian	Business woman	OND	Married	8	5
5	28	Yoruba	Christian	Chef	Bachelor's degree	Married	6	None
6	25	Yoruba	Christian	Student	Undergraduate	Married	6	None
7	30	Yoruba	Christian	Applicant	College of health	Married	5	2
8	19	Yoruba	Christian	Unemployed	Secondary school	Married	7	None
9	15	Yoruba	Christian	Student	Secondary school	Single	8	None
10	˃30	Igbo	Christian	Civil servant	Master's degree	Married	5	None
11	21	Yoruba	Christian	Applicant	OND	Married	7	4
12	33	Yoruba	Christian	Unemployed	HND	Married	8	None
13	31	Yoruba	Christian	Administrative officer	Bachelor's degree	Married	9	3
14	32	Edo	Christian	Civil servant	Bachelor's degree	Married	7	None
15	33	Yoruba	Christian	Trading	Bachelor's degree	Married	3	None
16	23	Yoruba	Christian	Trading	WAHEB	Single	8	None
17	25	Yoruba	Christian	CHEW	Tertiary	Married	7	2

NCE: national certificate in education; OND: ordinary national diploma; HND: higher national diploma; WAHEB: West Africa health examination board; CHEW: community health extension work

**Fears associated with childbirth:** more than half of the participants (12 of 17) said they had no fears whatsoever concerning childbirth. Few of the participants (5 of 17) mentioned that they had some fears about the childbirth/labour process. Participants were asked what fears they had concerning the process of childbirth. Some of their responses were: *“no complains…this is the first time, so I never pass through any type of delivery, pains or something like that.”*(P3, 27 years old, married). *“I´m not scared of anything. This is my first time now, I am a Christian and I´ve been talking to God, so no fear, I have that strong belief that I will deliver safely and have my baby and… no tears, hopefully no tears, no complication, no operation, cause I´m strong and I´m hoping for the best.”*(P5, 28 years old, married). Other responses included: *“I´m not scared of anything, but, because I know that God will take control, nothing at all.”*(P6, 25 years old, married). *“The delivery… I´m scared of the delivery.”*(P8, 19 years old, married). *“hmm, the fears… ah! I´m not… the only fear that I… although I didn´t count it as fear because I trust in God but the only thing that they use to say is maybe if the baby is big…they will…some people will have tears any other things. It´s only that one, but I believe in God that God will perfect everything.”*(P11, 21 years old, married). *“The pains and tears.”*(P14, 32 years old, married).

When participants were asked about the causes of these identified fears, out of the five participants who responded “yes” to having fears, two participants said they were afraid because of horror stories they´d heard concerning labour, two participants also said they were afraid because of the negative experiences of people they know who have gone through the labour process while one participant said she was afraid because due to the fact that she is too young. Some of the comments made were: *“Because I´m too small.”*(P8, 19 years old, married). *“I´ve heard lots of stories about it, so that´s the reason.”*(P14, 32 years old, married). *“Because of things that is happening around me, while some people labour maybe they are unable to give birth, maybe cause their pelvic is too small or someone, maybe they die during that process, but I pray that my own will not be that in Jesus name. So many things that is happen so that´s why I´m scared.”*(P16, 23 years old, single).

**Effects of fears associated with childbirth on preference for caesarean section:** when participants were asked if these fears can prevent them from getting pregnant again, of all five participants who responded “yes” to having fears, none of them admitted their fears are tangible enough to prevent them from getting pregnant subsequently. Some of the comments were: *“Ah! Anyway, I can´t say that o cause I´m…I plan to have child… I plan to have children, so now I know that I have to experience that pain too cause I´m a woman. So there´s no way I can escape it. So I´ve made up my mind that I´m going to go through the pains and I pray God will help me.”*(P17, 25 years old, married). *“Not at all.”*(P11, 21 years old, married). *“No, no, no, never. I still want to have children.”*(P14, 32 years old, married). “No! *laughs* no.”(P16, 23 years old, singe).

When asked if these fears are strong enough to cause participants to opt for a caesarean section, none of the participants said that these fears were strong enough for them to opt for a caesarean section. Some of the comments were: *“There is nothing that would make me want to have a caesarean section.”*(P16, 23 years old, married). *“No, not at all.”*(P11, 21 years old, married). *“Ah! I don´t think so, but at times if it happens, there is nothing I can do about it, but I didn´t pray to do caesarean section (CS). I´ll try my best to hold as long as I can bear the pains but if the doctors advise me that I should go for CS, that I can´t give the birth, I´ll have to agree with them because they know better than me.”*(P17, 25 years old, married).

**Preferred mode of delivery:** when asked if participants would mind undergoing a caesarean section if medically indicated, majority (8 of 12) said they would undergo a medically indicated caesarean section, few (3 of 12) said they would not while 1 participant was undecided. Some of their comments were: *“If it is compulsory, eh, I will do it. If that is to my advantage, I will go for it. That´s all.”*(P12, 33 years old, married). *“Sure! If hats the only procedure that they have to do to bring out my baby and me being alive too, my baby being alive, of course. Sure I´ll enter, I´ll go for it.”*(P10, above thirty, married). *“Accept it. Just for my baby to be alive and to save my life. There´s no problem.”*(P7, 30 years old, married). *“I can´t agree o, because even if they mention that kind of… that thing, I´ll just believe in God and continue to be praying that everything will come safely… cause most of my…em… like my sister now have never experienced… so I´ll just continue to be praying that God should do it.”*(P3, 27 years old, married). *“I can´t do it.”*(P1, 29 years, married). *“It´s only God that will take control. I can´t say if I will agree or not agree. I can´t say it.”*(P6, 5 years old, married).

When asked what mode of delivery participants think is best generally and reasons, of all 12 participants who said they had certain knowledge of caesarean section, almost all the participants (11 of 12) said they thought vaginal delivery was the best generally, only one participant said caesarean section is best, while one participant was for both sides. Some comments and reasons were: *“I prefer vaginal own. The only one reason that I know is that if deliver with vagina, is…any… you can still give birth, any… more than three or four times, but if it is CS, just three times, you cannot give birth again. After doing the CS… CS 3 times, you can´t give birth again.”*(P6, 25 years old, married). *“Ah! I think vaginal delivery is better o. Because that one you will experience the pain right? But after you´ve given birth, you´ll be okay, but if it is caesarean section, after some minutes that you´ve done the operation, you´ll still be feeling the pains, even some months. Some people, even some, like 3 months, they´ll still be feeling the pains, you know? It´s not the normal body again and it is also a scar for the body. Though I prefer vaginal delivery more than CS, but if there´s no option, I can go for it.”*(P17, 25 years old, married). “The normal one… it´s just giving birth by passing through the normal… vagina… because it reduces… after the sudden pain, the labour… after you´ve delivery, the pain is over, but you undergo any operation, you´ll still have to be nursing where the operation… the place of the operation and everything *hisses* so it´s better… once and for all and that´s it.”(P5, 28 years old, married). *“I think its CS though… because it´s less stressful and painful.”*(P14, 32 years old, married). *“Generally? In our Africa we think, uh, normal delivery… vagina… is the best for us in Africa, but if you´re to say it academical, CS saves pains, saves lot of things, saves your energy though you spend, it´s expensive, but there are some things that it will cover for you and if you´re operated by an expert, you´ll have a lot to gain concerning that, so there´ll be… they´ll protect your health, no complications, everything… they´ll do it perfectly and it doesn´t take anything than just to take care of the wound, that´s all.”*(P12, 33 years old, married).

When asked what mode of delivery participants prefer for current pregnancy and reasons, out of 12 participants who said they had certain knowledge of caesarean section, all (12 of 12) participants said they preferred to deliver vaginally for their current pregnancy. Some of the comments were: *“Vaginally…I´ve been with someone that gave birth through CS and I know what she passed through and I wouldn´t want that.”*(P10, above thirty, married). *“Yes ma. For this current pregnancy? Ah! I´ll have preferred normal delivery…vagina… because of the people around me, if they hear about CS, eh! it´s hell for me, but I´ll prefer normal delivery that´s all.”*(P12, 33 years old, married). “*laughs* I prefer vaginal delivery, because after some weeks, your body was normal, but CS, you cannot do CS and come back to normal again. As in your womb, your body, it cannot be normal again as normal delivery, that´s it.”(P15, 33 years old, married). *“Vaginal, because it´s natural.”*(P1, 29 years old, married). *“I´ll like to experience vagina.”*(P14, 32 years old, married).

## Discussion

Our findings revealed that majority (12 of 17) of the participants did not experience tocophobia while a few others (5 of 17) experience it, although these fear did not influence their birth choices. This may be because women within this geographical region (South-West, Nigeria) see childbirth as a normal phenomenon and something that should be experience by every woman. This may be because of the anticipation and excitement of bringing in a newborn into the world. This finding is in contrast with the results found in a study carried out in Indore, India where all (60 of 60) primigravid women were reported to have a severe level of fear related to childbirth [9].

Among the Few (5 of 17) participants who experienced tocophobia, tocophobia experience among these primigravid women were mostly associated with *“fear of pain”* and *“fear of vaginal tear”*. The present study findings are in support with a cross-sectional study that was conducted to explore the prevalence of FOC in Sweden, which concluded that childbirth related factors among primigravid women is based on fear of pain [10]. Concerning the origin/cause of the fears, one participant said she was afraid because she felt she was too small to give birth (19 years old) and this result is in contrast with that of a study conducted by Stella [9], where majority of the women who had significant level of fear were within the age range of 26-35 years. Furthermore, among the few participants who experience tocophobia, two of the participants said the fear must have been caused by *“horror stories”* they´d heard concerning labour and childbirth process, two participants also said they were afraid because of the *“negative/unpalatable experiences”* of relatives and close acquaintance. It can be deduced that the primary source of fear in this study was negative stories that they had heard about childbirth and unpleasant experience of acquaintance. These finding can be interpreted as a result of the fact that participants were primigravid women and did not experience childbirth before but heard frightening stories about childbirth from their relatives and friends. Findings were contrary to the findings of a study conducted by Hanna-Leena *et al*. 002 [7] which revealed that negative mood had the strongest relative explanatory power. Our findings however, was consistent, with the findings of a study conducted by Sercekus and Okumus [11] among Turkish women, where negative stories that women had heard about childbirth or health-care personnel was the primary source of fear.

Regarding the onset of fear, two participants confirmed that these fears had been brooding even before the realization of pregnancy while two participants confirmed the fears occurred during the pregnancy. Of all five participants who experience tocophobia, none of them admitted their fears are tangible enough to prevent them from getting pregnant subsequently. None of the participants said that these fears were strong enough for them to opt for a caesarean section. This may be because the women within this locale (Nigeria) have a negative perception towards caesarean section and viewed caesarean section as an abnormal way to deliver. This study is inconsistent with the study findings of Nieminen *et al*. [12], who found that there is a significant relationship between childbirth fear and women´s preference for caesarean section and also in contrast with a study conducted in a mid-Sweden county, where majority of the women who preferred and were delivered by caesarian section experienced tocophobia to a higher level compared to women with a vaginal delivery [13]. Childbirth fear in several studies have been reported as an effective cause in women´s preference for cesarean delivery, although this was not the case in this study.

It can be deduced that regardless of the fears had, all the participants in this study preferred vaginal delivery. Reasons for participants preference for vaginal delivery over caesarean section is because of a quicker post-delivery recovery and also because participants viewed vaginal delivery as a natural mode of delivery. This study is in agreement with the result of a study regarding delivery mode preference among Iranian women, [14] where women preference for vaginal delivery were associated with reasons such as “vaginal delivery was a healthier”, “more natural way of delivery”, “quicker labour recovery period” and “healthier postpartum” which is similar to what was obtained in this study. Similar findings were reported in a study carried out in Egypt [15], where about two thirds of women preferred vaginal delivery due to quicker post-delivery recovery, and their perception that vaginal delivery is a natural way of delivery. This view/perception may be due to participants´ culture. In a study conducted in Ebonyi State University Teaching Hospital Nigeria (South-Eastern region of Nigeria), in 2011 [16], most women viewed caesarean section as an abnormal way/mode of delivery. Similar feelings (perception) were reported by Okonkwo *et al*. 2012 [6] where women majority (85.2%) of the women who had undergone a previous caesarean delivery, said they would like to experience vaginal delivery in their next pregnancy in order to feel like “a real woman”. This further affirms, the importance/premium women in this part of the country (South-West Nigeria) places on vaginal delivery, which is not uncommon in the African culture.

All the participants in this study expressed strong optimism of being able to go through the delivery process. This optimism seem to be inclined to their “belief in God” who they belief could help them through the birthing process. Similar belief in God were expressed by Ghanaian women in a study conducted by Aziato *et al*. 2016 [17] where women prayed to God to ensure a successful delivery and prevent misfortunes or activities of evil spirits that affect the outcome of pregnancy. This suggests that women in this study had faith in God. This assertion is also supported by Hancock *et al*. 2013 [18].

**Limitation of the study:** due to the small sample size and the purposive sampling used in selecting study participants (antenatal women) in a tertiary health care facility in Nigeria, hence the results may not be generalizable to a larger context.

## Conclusion

This study demonstrated that only a few of the primigravid women in Ekiti State have tocophobia experience and these fears have no effect on their preference for caesarean section as all the participants prefer to deliver through vaginal delivery. It has been observed from this study that there is a negative perception towards delivery through caesarean section.

**Implications of findings for practice:** fear of childbirth has effects on women´s health as it is a major psychological issue that promotes women´s requests for medical interferences and interruption of physiological labour. It is therefore important for midwives to be aware of their roles in counselling and identifying early women with tocophobia during focused antenatal care, so as to promote healthy pregnancy outcome.

### What is known about this topic

- Studies have shown that fear of childbirth affects about 20% of women in the world;
- Certain levels of fear and anxiety about childbirth are expected, especially among primigravid mothers;
- These feelings may become intense as to negatively influence a woman´s decisions and perceptions about the birth process.

### What this study adds

- Tocophobia in this study was associated with fear of labour pains and fear of vaginal tear;
- Reasons for this fear (tocophobia) was majorly attributed to negative experiences of people/mothers in the neighborhood and frightening stories of childbirth told by relatives and friends;
- Although tocophobia experience of participants did not affect their birth choices, as all the participants preferred vaginal delivery over caesarian section. It is important for midwives to identify early antenatal women with tocophobia, so that appropriate interventions could be put in place for good and safe transition to motherhood.
